# An Investigation of the Needs of Saudi Parents of Preterm Infants in the Neonatal Intensive Care Unit

**DOI:** 10.7759/cureus.3887

**Published:** 2019-01-15

**Authors:** Eman M Alsaiari, Judy Magarey, Philippa Rasmussen

**Affiliations:** 1 Pediatrics, Prince Sattam Bin Abdulaziz University, Riyadh, SAU; 2 Pediatrics, The University of Adelaide, Adelaide, AUS

**Keywords:** parents, preterm infants, needs, nicu, saudi

## Abstract

Objective

This study aimed to identify the needs of Saudi parents who had an infant in a neonatal intensive care unit (NICU) in one of five hospitals in Riyadh City, Saudi Arabia.

Materials and methods

Data were collected using a questionnaire that comprised questions about demographic characteristics and a modified version of the NICU Family Needs Inventory. A convenience sample of 36 Muslim Saudi mothers and fathers completed a self-reported questionnaire. Parents were asked to rate 52 statements in the NICU Family Needs Inventory as not important, somewhat important, important or very important.

Results

Saudi parents ranked the needs for assurance, proximity, and information as the most important needs. The comfort and support needs were ranked as the least important. Moreover, the highest top-ranked items were related to assurance of pain infant being treated for (86%), infant expected outcome (83%), and infant being handled gently (83%).

Conclusion

Nurses should create a relationship with parents and provide them with comprehensible and honest assurance and information. Likewise, it is imperative to provide a high-quality holistic care for parents that relies on their needs assessment.

## Introduction

A preterm infant is a baby born at less than 37 weeks of gestation [1]. These infants are generally admitted to a neonatal intensive care unit (NICU) because their underdeveloped condition renders them vulnerable to life-threatening diseases, such as sepsis, aspiration, malfunctioning digestive systems and respiratory failure [1].

Birth of preterm infants and hospitalization in NICU is a stressful experience for parents worldwide [2,3]. Parents of preterm infants' experience multiple stressors related to several adverse conditions such as, the preterm birth, postpartum mother’s medical state, new parenthood feelings, vulnerability status of the infant and infant physical separation due to admission to NICU [4,5]. Further stressors include as well the arranged and controlled opportunities of parents’ interaction with their infants, inability to support infant’s care, fear of infant survival and development, the intensified feelings of love, hope, fear, and loss [2,6]. NICU environment, facilities and technology, unpleasant machines sound, medical procedures, and rounds of health care providers are a source of stress as well [7]. These stressors adversely affect parents physically, psychosocially and spiritually, and can affect the relationship between parents and their infant [8].

Parents of preterm infants have indicated the significance of obtaining infants’ health information and guidance [9], trusting medical staff [10], and experiencing support from staff to meet their needs [10,11]. Therefore, it is awfully important for NICU nurses to support parents and provide holistic and comprehensive care to them by recognizing and meeting parents’ needs [12] to relieve or diminish their distress or improve their sense of adequacy [13]. If their parnets' needs are not met, they are likely to become more stressed and even less able to cope.

Parents in both Western and Eastern Muslim cultures reported the need for proximity, regular information related to their infant’s health and regular communication with NICU nurses. They reported that meeting these needs has helped them in reducing the intensity of stress and associated negative feelings and in increasing their boding and attachment to their infant [9,14-19].

There is a scarcity in the literature investigating the needs of Saudi parents who have preterm infants hospitalized in the NICU. This is an important gap in the literature because the needs of Saudi parents with preterm infants in the NICU in Saudi Arabia are not the same as those in Western countries. The culture and characteristics of parents and infants are different from one setting to the other [4].

Through an understanding of the needs of Saudi Arabian parents who have a preterm infant in a NICU, health care professionals will be well prepared to meet parents’ needs by providing the appropriate support and help. This may reduce parents’ stress, and may, in turn, lead to better health outcomes for both parents and infants. Therefore, we aimed in this study to explore Saudi parents’ needs of hospitalized preterm infants in NICU.

## Materials and methods

Study design and settings

Cross-sectional study design was conducted during March 2014 in five hospitals in Riyadh city namely, King Fahd Medical City, King Saud Medical City, Al-Yamamh Hospital, Aleman Hospital, and Prince Salman Hospital.

Study subjects and recruitment

A convenience sample of Saudi parents visiting their infants who were hospitalized in levels II and III NICUs was identified and invited to participate in the study. Parents with preterm infants and still hospitalized in NICU at the time of the study were considered eligible to take part in the study. Parents of full-term infants admitted to the NICU were excluded because the survey was believed not to be appropriate for them.

Parents were recruited by the NICU bedside nurses who gave the study information sheet to eligible parents and asked them to read it. Then, interested parents who agreed to participate were given the questionnaire to complete.

Data collection and instrument

Data were collected using a tailored self-administered questionnaire that comprised questions about demographic characteristics and a modified version of the NICU Family Needs Inventory. The demographic section captured parents’ demographics: age, relationship to the infant, marital status, number of children they have, previous experience of having an infant in a NICU, level of education, occupation, income, and number of visits to their infant in the NICU. Also, the demographic section collects data about the infant’s age, length of stay in the hospital, level of NICU care and medical diagnosis.

Parents’ needs inventory

A tailored version of the NICU Family Needs Inventory [20] was used for exploring parents’ needs of preterm infants. The NICU Family Needs Inventory is divided into five domains: ‘support (interpersonal and emotional needs); comfort (personal and physical comfort); information (pragmatic information about the infant); proximity (remain in the range of infant); and assurance (experience confidence about the infant’). The NICU Family Needs Inventory comprised of 56 statements to measure the importance attributed to parents’ needs using a 4-point Likert scale (not important, somewhat important, important and very important) for parents to rate their response to each statement.

The following three items were excluded: ‘to be able to visit my infant at any time,’ ‘to see my infant frequently,’ and ‘to have a private place to breastfeed or use a breast pump.’ The first two items were excluded because the NICUs at the participating hospitals are already open 24-hours a day for parents to visit their infants. The third item was excluded because the NICUs of the participating hospitals already had private rooms for breastfeeding.

One statement, ‘to have a pastor, clergy, or other person from my church visit’ required modification to reflect the culture and religion of Muslim parents in Saudi Arabia. After modification, the statement read: ‘to allow a religious person to visit and do Roquia for my infant’.

One statement was added, ‘To have prayer room near the NICU’. The statement was added because there are currently no prayer rooms near the NICUs of the participating hospitals. This statement is an important addition because Saudi parents need to be able to practice their spiritual rites near their infant.

Linguistic adaptation: the English version of the modified questionnaire was translated forward into Arabic and then backward from Arabic to English by the study author. Then, a panel of two bilingual experts in translation was asked to check the accuracy of the translation. Few corrections were required. Grammatical mistakes in items 5, 3 and 11 and a few words in items 22, 25 and 40 were changed to make the meaning clear for the reader. The differences between the two bilingual experts were elucidated by consensus and combined into the Arabic version.

Reliability and validity of the modified NICU Family Needs Inventory

The face and logical content validity of the modified tool were assessed by two NICU head nurses working in two different hospitals in Riyadh. The two NICU head nurses agreed that all the items were essential and no additional statements were necessary and found the content of the instrument to be valid. The overall value of Cronbach’s alpha for the 51 items modified NICU Family Needs Inventory was 0.95.

Statistical analysis

A descriptive analysis was used to describe parents’ demographics and infants’ clinical characteristics. The mean and standard deviation were used to compare between the five sub-scales (Information, Proximity, Assurance, Comfort, Support). ANOVA test was used to find out the difference between the sub-scales.

Ethical considerations

Before the study conduct, written IRB approvals were obtained from the University of Adelaide’s Human Research Ethics Committee (the General Administration of Medical Research in the Saudi Arabian Ministry of Health). Participants' identity was kept confidential. Parents’ completion of the questionnaire was considered implied consent.

## Results

A total of 36 parents (23 mothers and 13 fathers) participated in the study with a mean age 28.44 ± 6.31. The majority 15 (44.1%) of the parents were at university level of education and have <10.000 USD annual income. Furthermore, over one-quarter of the parents do not have other children and 16 (44.4%) have only one child. Slightly more than three-quarters reported they do not have previous experience of having a preterm infant in the NICU. Thirty percent reported a daily visit to the NICU (Table 1).

**Table 1:** Parents’ characteristics (n = 36). NICU: Neonatal intensive care unit

Gender	
Mothers	23 (63.8%)
Fathers	13 (36.2%)
Age	28.44 ± 6.31
Marital status	
Married	34 (94.4%)
Widowed	2 (5.6%)
Level of education (n = 34)	
Primary	4 (11.8%)
Secondary	4 (11.8%)
High school	11 (32.3%)
University and above	15 (44.1%)
Annual income (USD)	
<10.000	23 (63.9%)
10.000-20.000	9 (25%)
20.001-30.000	1 (2.8%)
30.001-40.000	3 (8.3%)
Occupation	
Housewife	17 (53.1%)
Nurse	2 (6.3%)
Government service	2 (6.3%)
Business	4 (12.5%)
Teacher	7 (21.8)
Number of other children	
None	10 (27.8%)
1	16 (44.4%)
2	2 (5.6%)
3	5 (13.9%)
4	3 (8.3%)
Previous experience of having preterm infant in the NICU	
Yes	8 (22.2%)
No	28 (77.8%)
Frequency of parental visits to the NICU	
Daily	30 (85.7%)
1-2 days a week	1 (2.9%)
3-6 days a week	4 (11.4%)

Table 2 shows that the infant's current age at the time of the study (days) was 32.51 ± 58.23 and their mean length of stay in the NICU was 27.56 ± 46.34. Table 2 lists the infants’ health problems for NICU admission. Besides prematurity (13, 36.1%), seven (19.4%) of the infants were diagnosed with respiratory problems, followed by undetermined diagnoses (8, 22.2%), heart problems (3, 8.3%) and low birth weight (3, 8.3%). Over half of the infants were under level I and 13 (37.1%) were under level II.

**Table 2:** Infants’ clinical characteristics. NICU: Neonatal intensive care unit

Infants’ current age (days)	32.51 ± 58.23
Infants’ length of stay in NICU (days)	27.56 ± 46.34
Infants’ health problems	
Premature only	13 (36.1%)
Premature with respiratory problems	7 (19.4%)
Premature with low birth weight	3 (8.3%)
Premature with heart problems	3 (8.3%)
Premature and jaundiced	2 (5.6%)
Premature plus undetermined diagnoses	8 (22.2%)
Level of care (n = 35)	
Level I	19 (54.3%)
Level II	13 (37.1%)
Level III	3 (8.6%)

The most important parents’ needs

Table 3 shows that items from the assurance scale dominated the highly ranked ‘very important’ and ‘important’ items. Six of these 11 items that were ranked highest regarding the number of ‘very important’ responses were from the assurance scale. The highest three ranked items ‘to know that infant was being treated for pain’ at 86%, ‘to know the expected outcome’ at 83%, and ‘to know that infant was being handled gently’ at 83%.

**Table 3:** Most important parents’ needs of infants in NICU. NICU: Neonatal intensive care unit

Parents’ needs	n (n%)
To know that infant being treated for pain	31 (86.1%)
To know the expected outcome for my infant	30 (83.3%)
To know that my infant being handled gently by health care provider	30 (83.3%)
To be called at home about important changes	29 (80.6%)
To be assured of the best care possible is being given to my infant	28 (77.8%)
To receive information about infant at least once a day	28 (77.8%)
To feel there is hope	28 (77.8%)
To know how infant being treated medically	28 (77.8%)
To see NICU staff provide comfort to my infant, such as giving my infant pacifier, using blanket to support infant body and talking softly to my infant	28 (77.8%)
To know specific facts concerning progress	27 (75%)
To have questions about infant answered honestly	27 (75%)

The least important parents’ needs

Table 4 shows that most of the items ranked least important by parents came from the support scale; where five of the eight items reported as ‘not important’ came from this scale. Those needs are ‘to have a support group of other families’ at 25%, followed by the items ‘to have a place to be alone’, ‘to have a place to sleep’, and ‘to have friends/family nearby’ at 22% for all. The final items included one from the proximity scale and two from the information scale. Those items were ‘to have waiting room near NICU’ (proximity scale) at 19.4%, and ‘to feel it is alright to cry’ at 19.4%, and ‘to receive help in responding to reactions of infant siblings’ at 16.7% (information scale).

**Table 4:** Least important parents’ needs of infants in NICU. NICU: Neonatal intensive care unit

Needs	n (n%)
To have a support group of other families available	9 (25%)
To have a place to be alone	8 (22.2%)
To have a place to sleep near the NICU	8 (22.2%)
To have friends/family nearby	8 (22.2%)
To have waiting room near the NICU	7 (19.4%)
To feel it is alright to cry	7 (19.4%)
To receive help in responding to reactions of infant siblings	6 (16.7%)
To have classes about premature infants special care needs	6 (16.7%)

NICU Family Needs Inventory subscales for parents

Overall, the highest reported mean score was for the assurance subscale (3.62 ± 0.72), and the least reported subscale was for the support (3.13 ± 1.07), with a statistically significant difference among the subscales (p < 0.001) (Table 5).

**Table 5:** Mean and SD of NICU Family Needs Inventory subscales. NICU: Neonatal intensive care unit

Scale	Mean ± SD	p-value
Support	3.13 ± 1.07	<0.001
Comfort	3.25 ± 0.97	
Information	3.37 ± 0.90	
Proximity	3.48 ± 0.83	
Assurance	3.62 ± 0.72	

## Discussion

The current study aimed to explore Saudi parents’ needs of hospitalized preterm infants in NICU. The results of the study showed that the parents viewed needs related to assurance and proximity as most important, followed by information needs. Support and comfort needs were viewed as least important.

Interestingly, the ranking of the subscales of family needs was similar to previous studies that have reported similar findings and revealed that assurance was the most important need [4,13,20]. Moreover, parents in this study ranked support needs as the least important subscale need [4,13,20].

Although parents participated in this study ranked information needs as the third most important need, the information needs were reported in the literature as the most important family need [21-23]. The reassurance items were reported in the literature among the top 10 parents’ needs [4,13,20].

The ranking of assurance as the most important need supports the conclusions drawn by researchers in several qualitative studies that examined the lived experience of Eastern-Western parents of infants in the NICU [15,16,24,25]. Parents in those qualitative studies reported the experience of having a preterm infant was overwhelming, they have a significant level of anxiety and fear about their infants’ outcome, and understandably, they reported fears about losing their infant and the possibility of having a child with serious health problems and disability. Those feelings decrease gradually when their infant’s condition improves [15,16,24,25]. In this study, parents of infants in NICU experienced a significant amount of stress, anxiety and fear about their infant’s condition and not knowing if their child was going to recover or not or going to recover with a severe health problem. Therefore, it is not surprising that the parents need to be reassured that their infants were receiving high-quality health care.

In this study, over half of the infants were in level 1 NICU, which means that they are under a high level of medical care and parents have limited proximity to their infant. This has also been evident in several studies that examined the experience of parents who have infants in a NICU. Those parents found that their proximity was limited when their infant received a high level of intensive care [16,24]. Proximity can also be limited by the long period of infants’ hospitalization in the level 1 NICU. The mean length of level 1 NICU hospitalization for preterm infants is 27.58 days. This long period of separation between parents and their infants increases the desire of parents to be close to their infant. Another factor that has been reported by parents in the literature is that it was stressful for them when leaving the infant at the hospital to be at home with their other children. This has given parents feelings of guilt because they are not fulfilling their parental role toward their sick infant, and this has increased their desire for proximity. For instance, fathers experienced significant stress, feelings of guilt and a lack of control as a result of not fulfilling their parental role to be near their infant and partner in the hospital. The inability of fathers to be near their infants and partners was the result of difficulties in managing their time to be able to visit their infant in the hospital while also attending to their home and work responsibilities [9,17]. Another reported interpretation is the nurses’ behavior could limit proximity during parents’ visiting times. Mothers reported the desire to be close and involved in their infant’s care, but nurses could consider their involvement as a hindrance to their work, or label the mother as a troublemaker, and this could be a barrier to meeting the mothers’ needs for proximity. Mothers reported that the workload of nurses could mean that they are unable to meet the proximity needs of parents [16,24,25]. Therefore, after consideration of the above factors, it is not surprising that Saudi parents have ranked the need for proximity second after the need for assurance.

The third scale that was ranked by Saudi parents was information. This result was not similar to those of previous studies conducted on parents of neonates. In Ward’s (2000) and Nicholas’s (2006) and Mundy’s (2010) studies [4,13,20], parents ranked information as the second most important need. Their results could be attributed to the time of study conduct. During the first week of infant hospitalization, parents need information regarding their infant’s condition. For this study, the need for information was still considered important but was ranked third among the subscales. One explanation for this finding is the length of infant stay in the hospital. This could be supported by the conclusions of Nicholas (2006) [4], where the longer the infant was at the hospital, the less emphasis on asking for information. Another explanation could be related to the frequency of visits. In this study, the majority (N = 30; 85%) of Saudi parents visited their infant daily and were provided with daily updates with information regarding their infant condition.

Comfort and support were ranked at the fourth and fifth least important needs. It may be that, due to the child’s crisis, the parents cannot recognize that they have support and comfort needs that can be as important as the needs of their infant. It has been shown that parents want staff to focus on their infant’s health and preserve his or her life and that parents sacrifice their needs by rating them as low importance [26]. Another interpretation for support as the lowest ranked need is that the NICU unit where the study took place did not offer a support program for families who had infants in the NICU. The low parental ratings of support could be because they were unable to participate in a support group [20]. Another consideration is that Saudi families have strong ties and a mutual commitment between family members. This includes visiting ill relatives and their families to provide emotional, psychological and financial support. This can also be explained from the perspective of Saudi Islamic culture because this type of support can be provided to the patient and immediate family by the extended family.

Limitations

Sample bias may have arisen through the use of a convenience sample, and this limits the generalizability of the findings for two reasons. There was a low response rate because only those who were interested in the study completed the questionnaire. Also, there were more females in the sample than males, and this might not represent the target population. The results of this study did not capture the opinions of parents who did not visit their infants frequently enough to be approached to participate in the study. This group of parents may have very different opinions about the needs they consider being the most important. Non-respondent bias may have arisen because, in some sections of the questionnaire, the participants have not all provided answers. This could affect the validity of the study’s result. This could be related to parents who were under a lot of stress because of their infant’s condition and it was hard for them to understand and answer each part of the questionnaire.

## Conclusions

The results of our study reported the needs for assurance, proximity and information were found to be most important to Saudi parents. Support and comfort needs were found to be least important needs. The characteristics of parents and infants, the culture of the parents and the current behaviour of nursing staff have all influenced how parents ranked their needs. The findings from this study can be used to inform health care providers with insight to assist in implementing successful support programs to meet parents’ needs.
